# Supplementary material for: Trends in hyperinsulinemia and insulin resistance among nondiabetic US adults, NHANES, 1999–2018
Source: Res Sq. 2024 Nov 18:rs.3.rs-5279795. Preprint. [Version 1] doi: 10.21203/rs.3.rs-5279795/v1 (PMC11601873; doi:10.21203/rs.3.rs-5279795/v1)
Supplement: Supplement 1 [file NIHPPRS5279795v1-supplement-1.pdf]

Supplementary Files

This is a list of supplementary files associated with this preprint. Click to download.

- supplement.docx
